# Nutritive Value of Wheat Meal

**Published:** 1879-10

**Authors:** 


Nutritive Value of Wheat Meal.—*The London Dietetic Reformer* shows, by scientific data, that wheat meal, which is cheaper than bolted meal or fine flour, contains one third more nutriment than flour does, from which the bran has been sifted. Fine flour, according to this journal, is not food at all, in the proper sense of the term—that is, the elements of the grain which are separated in the process of bolting, being essential to perfect nutrition, those who use fine flour are obliged to subsist mainly on other things, or lose their health; that no one, therefore, who makes baker's bread a principal article of diet can long maintain health, while those who use wheat meal bread, unfermented and unadulterated, can maintain their health with a very small addition of other foods.

